# Health policy experts’ perspectives on implementing mental health specialist video consultations in routine primary care – a qualitative interview study

**DOI:** 10.1186/s12913-021-06676-x

**Published:** 2021-07-20

**Authors:** Justus Tönnies, Lydia Oeljeklaus, Michel Wensing, Mechthild Hartmann, Hans-Christoph Friederich, Markus W. Haun

**Affiliations:** 1 Department of General Internal Medicine and Psychosomatics, Heidelberg University, Im Neuenheimer Feld 410, 69120 Heidelberg, Germany; 2 Department of General Practice and Health Services Research, Heidelberg University, Im Neuenheimer Feld 130.3, 69120 Heidelberg, Germany

**Keywords:** Health policy, Mental health, Primary care, Integrated care, Telemedicine, Video consultations, Videoconferencing, Implementation, Thematic analysis, Qualitative research

## Abstract

**Background:**

Many patients with mental disorders are treated by their general practitioner (GP). Innovative technology-based integrated care models (e.g., mental health specialist video consultations) have been proposed to facilitate access to specialist services in primary care settings. While perspectives of patients and providers have been examined, there is little insight into the perspectives of health policy experts on such models. The purpose of this study was to examine the perspectives of health policy experts on (1) current challenges for continuity of care, (2) anticipated benefits and barriers for implementation of mental health specialist video consultations along with (3) practical and regulative preconditions for sustained implementation in primary care.

**Methods:**

In a cross-sectional qualitative study, we conducted 15 semi-structured interviews with health policy experts representing various stakeholders in the German health care system: health insurances, governmental bodies, clinicians’ professional associations, and patient representatives. Following a critical realism approach, we applied a qualitative inductive content analysis to derive key themes from the material.

**Results:**

Health policy experts saw long waiting times for patients and a lack of collaboration between in- and outpatient mental health services as well as mental health specialists and GPs as main barriers for current continuity of care. Health policy experts also felt that video consultations bear great potential to foster coordinated care between GPs and specialists and ensure timely referral for severely burdened patients. Increased workload for the general practice staff to facilitate video consultations and difficulties in establishing reliable therapeutic alliances between patients and specialists via remote treatment were considered as major barriers. Health policy experts varied significantly in their level of knowledge concerning legal frameworks and regulations pertaining to video consultations. However, the implementation of appropriate reimbursement schemes and sufficient data protection were regarded as the major regulative challenges.

**Conclusions:**

Health policy experts mostly consider mental health specialist video consultations as a promising way to overcome current challenges for the management of patients with mental disorders at the interface between primary and specialist care. To ensure sustained implementation, a multi-stakeholder approach accounting for the perspective of health policy experts, patients, and providers should be followed.

**Trial registration:**

German Clinical Trials Register DRKS00012487

**Supplementary Information:**

The online version contains supplementary material available at 10.1186/s12913-021-06676-x.

## Introduction

Mental health conditions are significant public health problems and are associated with a substantial global and individual disease and economic burden [1, 2]. General practitioners (GPs) provide comprehensive care to most of their patients suffering from such conditions and who mostly prefer to be cared for by their GP [3, 4]. However, the integration of specialized mental health care may be required for optimal treatment effects, as it improves patient outcomes, entails more timely care for patients, fosters medication adherence, increases continuity of care and may also reduce long-term costs by averting more intensive treatment and incapacity to work [5–9]. Reasons for the limited access to mental health specialists are multi-fold and include, for instance, long waiting times [10, 11]. Furthermore, increasing rates of multimorbidity due to demographic change in high-income countries contribute to a substantial proportion of patients remaining with their GP [12, 13].

Integrated mental health care models have been shown to ensure a more seamless trajectory between primary and specialised care [8, 14–19]. However, since these models are not practical in rural and remote areas, they were extended by real-time video consultations conducted by mental health specialists to overcome geographical barriers [20–22]. Several randomised-controlled trials primarily addressing depression, anxiety, and posttraumatic stress disorder show integrated video-based treatment to be equally effective, safe and potentially cost-effective compared to same-room treatment by saving travel times for patients [23–28]. Furthermore, there is a fairly large body of evidence of acceptance and intent to adopt these new service delivery models [23, 29–31].

Since these approaches were developed and evaluated in the US, their usefulness in other countries and healthcare systems need to be examined. Many European health care systems are publicly funded and have low financial barriers to healthcare. In contrast, in the US system health insurance is not mandatory or a national affair and two thirds of the US citizens are privately insured [32]. Furthermore, in many European countries especially in France, the Netherlands, Italy, and Germany the average number of physicians per general practice who constitute a key role in integrated mental health care is lower than in the US [33]. Consequently, they have fewer resources for employing additional staff such as mental health specialists.

It is essential to have an insight into micro level (patients and health care provider), e.g., usability, intent to adopt, acceptability, as well as into macro level perspectives (health policy experts), e.g., policy and legal barriers and facilitators, reimbursement schemes, data protection. At the macro level, with respect to sustained implementation of eHealth models, it is necessary to take legal frameworks, existing, partly fragmented, and changing regulations and realistic financing outside a research project into account.

Interview studies, conducted in Germany and other countries (mainly the USA) as well as a literature review exploring the perceptions of different stakeholder groups (patients, health care providers, and health policy experts) regarding the introduction of eHealth models revealed some of the main constraints and facilitators that affect the implementation of such models at the macro level [34–37]. However, these studies report data on barriers and facilitators for the implementation of innovative health care models in general and do not specifically concern these aspects regarding mental health specialists video consultations.

To get a comprehensive overview on prior work in this specific field, we conducted a systematic search in MEDLINE (Additional file 1). Among the 598 records, we found two records concerning health policy experts’ perspectives on eHealth interventions for mental health care. One study originates from Australia and summarizes the discussion at a multidisciplinary conference on technology supported innovations for mental health care [38]. The authors identified several barriers to implement technology-supported interventions in mental health care which were categorised as either structural issues surrounding mental health policy and services in general or technology-specific issues. The latter were concerns how to translate therapeutic principles to a technology-based medium, reservations about data protection, and the resulting lack of trust in eHealth interventions.

The other study was an online survey conducted in several European countries on stakeholders’ opinions towards digital treatment for depression [39]. In that survey, 175 stakeholders participated and highlighted reducing costs as the main benefit from such interventions. Various barriers were identified and related to technology infrastructure, lack of effectiveness, and negative attitudes from patients and providers.

Although there is an overlap in terms of perceived barriers [40], the disparities between different stakeholders must be considered. Additionally, since we did not find any studies that specifically focussed on the implementation of video consultations in mental health care, there is still a lack of information how health policy experts assess mental health specialists video consultations as an addition to conventional mental health care settings. Consequently, to account for system-related differences and the implementation of specific eHealth interventions such as mental health specialists video consultations into routine care research projects should address barriers at multiple levels and follow a multi-stakeholder approach.

The PROVIDE project (Im**pro**ve cross sectoral collaboration between primary and psychosocial care through implementing **vid**eo consultations in primary care) aims to develop, evaluate and implement mental health specialists video consultations to expand the care of patients with mental health problems with an online real-time treatment offer [41–44]. The purpose of this pre-implementation study was to examine the perspectives of health policy experts on (1) current challenges for continuity of care, (2) anticipated benefits and barriers of mental health specialists video consultations along with (3) practical and regulative preconditions for their sustained implementation into primary care.

## Methods

### Study design and conceptual framework

In a naturalistic qualitative explorative study, we conducted one-off semi-structured problem-centred interviews [45]. To account for an inherent subjectivity in the production of knowledge, we chose a critical realist position for designing the study, analysing the data, and interpreting the results [46]. Specifically, we assumed social structures independent of our understanding (e.g., technology commitment in health providers) and examined how participants constructed meanings when engaging with these structures (e.g., what does technology commitment entail for the respective individual) and in doing so aimed to account for our own experience and background as researchers (e.g., how do clinician-scientists influence technology commitment in health providers). This study was approved by the Ethics Committee of the Medical Faculty at Heidelberg University (no. S-197/2017) and preregistered with the German Clinical Trials Register (registration no. DRKS00012487). We obtained written informed consent from all participants prior to study enrolment. We followed the consolidated criteria for reporting qualitative research (COREQ) guidelines for reporting the study results [47].

### Study setting

The study was conducted in Germany where statutory health insurance, handled by nongovernmental insurers known as sickness funds, provides inpatient, outpatient, mental health, and prescription drug coverage for approximately 86% of the population (for more details please see: https://www.commonwealthfund.org/international-health-policy-center/countries/germany). GPs are reimbursed through regionally negotiated fee-for-service payments up to maximum number of services per quarter. There is generally no gatekeeping and patient registration is not required (free-access system), but sickness funds are required to offer the option to enrol in a family physician model with gatekeeping. In the German health care system, integrated mental health care models have rarely been implemented so far.

### Participants and recruitment

Following a purposive strategy to select information-rich cases with characteristics that are rare and difficult to find, we applied non-discriminative snowball sampling where a recruited subject provides multiple referrals [48]. Each new referral was explored until sufficient theme saturation was reached. Based on a report by the Agency for Healthcare Research and Quality Scientific Resource Center Working Group we categorised the health policy experts into five expert groups, namely representatives of government agencies, payers, clinicians and representatives of their professional associations, patient representatives, and health care policy makers at federal, state, and local levels (see Table 1) [49]. This leads to a better comparability with other studies that have interviewed health policy experts before.

**Table 1:** Classification of stakeholders in expert groups

Expert groups	Description	Number of interviews conducted
Representatives of government agencies	Representative of the Federal Ministry of Health	1
Payers	Consultants and project manager of health insurance companies	3
Clinicians/representatives of their professional associations	Representatives (chairmen/women) of national and federal medical and psychological/psychosomatic associations and state chambers	8
Patient representatives	Representative of association for patient self-help groups	1
Health care policy makers at federal, state, and local levels	Representatives (project manager and board member) of the Association of Statutory Health Insurance Physicians Baden-Wuerttemberg	2

Note: description of the five expert groups and the number of participants per group

### Data collection

We conducted 15 individual interviews (two of them in the health policy expert’s office, 13 via telephone) (median: 51.3 min, interquartile range: 6.1 min). The last author (male, MD, attending physician in psychosomatics and psychotherapy, senior researcher with > 5 years of experience with qualitative research) and content expert for mental health services, conducted the interviews assisted by Mariell Hoffmann (MH, female, master’s degree in sociology). They had no relationship with any participant prior to the study, introduced themselves as researchers to the participants and guaranteed the absence of nonparticipants during the interview. All interviews were audio recorded, and we supplemented the audio data with field notes produced during the interviews. To capture health policy experts’ descriptions systematically, we designed a semi-structured interview guide (Additional file 2) that was reviewed after the first three interviews. Since the interview guide proved to be coherent and comprehensive, no changes were made. First, we asked participants about their perception of the current continuity of care for patients with mental health conditions. Second, we introduced the intended model, including that the patient would be located in the primary care practice while the mental health specialist would consult from her or his office or private practice or a suitable, designated room at home. Finally, we asked about anticipated benefits and barriers along with regulative and legal preconditions for the implementation of mental health specialists video consultations. In between the interviews, we discussed the progress of sampling and data collection, e.g., with respect to referrals from the snowball sampling and the level of data saturation [50]. Qualitative data uploaded to a secure server of Heidelberg University Hospital, which was accessible only to the research team.

### Data analysis

After the audio recordings were transcribed verbatim by a professional transcription service (Transkripto, Rotterdam, Netherlands), we anonymized the data. We did not return the transcripts to the participants for comments. First, two coders (Lydia Oeljeklaus (LO), female, master’s degree in psychology, research assistant, assisted by Mariell Hoffmann) independently conducted a computer-assisted thematic analysis in MAXQDA Analytics Pro 2020 (VERBI GmbH) of three interviews, i.e., 20% of the entire transcript material [51]. The coders initially followed an inductive (bottom-up) approach by paraphrasing, generalizing, and abstracting the original data. Both coders compared their analyses and resolved disagreements in a final code system. Generally, we transformed data from the coded segments to the results by writing by thematic summaries using summary grids. Specifically, for each case, the segments coded with a specific category were brought into focus (What did person A say about the topic “Anticipated Benefits from and Barriers for Implementing Mental Health Specialist Video Consultations” during the course of the respective interview?). Using MAXQDA’s Summary Grid function we compiled all these places in the interview and then wrote a thematic summary for all of them combined. Second, we applied this code system to analyse the remaining 13 transcripts top-down. We discussed newly derived themes and modified the codes when necessary, to ensure that all key aspects were represented. We felt that theme saturation was reached when the analysed data did not provide any new themes or meaning of themes, that is, when the inductively developed themes represented and covered all the data [50]. Additional file 3 provides an overview of the key themes, including definitions and supporting quotes.

## Results

### Current challenges for continuity of care

Three main issues related to macro level of current care provided for patients with mental disorders were perceived. First, all expert groups criticised regulations that mainly refer to waiting times and an insufficient number of available mental health specialists. The waiting times for mental health care were considered to be too long.

*“The waiting times are too long, for various reasons. And this is also true in the formally overserved areas.” (clinician/representative of his/her professional association)*

Additionally, when it comes to the treatment for acutely and severely stressed patients, mental health specialists’ availability and resources are also very limited.

*“This [the insufficient treatment of severely burdened patients] has to do with the fact that there is not always enough time during a therapy session available for guideline-compliant treatment in the setting of a single practice.” (representative of a government agency)*

Second, all expert groups mentioned barriers through insufficient collaboration. Especially the strong separation between outpatient and inpatient treatment of patients with mental disorders hampers intersectoral and cross-professional care and leads to conflicts between providers (e.g., patients in home-treatment were cared by inpatient providers instead of office-based psychiatrists). In addition, the absence of networks between mental health specialists and GPs lead to insufficient treatment of patients with mental disorders.

“*However, it is not only about demand planning for the outpatient sector, but also regulation in the inpatient sector and in the cooperation between the two sectors. The difficulty of working across sectors [...]. Even if someone wants to participate in it, in concrete terms it is made more difficult or even prohibited.” (clinician/representative of his/her professional association)*

Finally, a restriction in the equity of care was mentioned by almost all expert groups (except of health care policy makers at federal, state, and local levels). In particular, older and severely burdened patients with mental disorders would have significantly lower chances to receive adequate care.

### Anticipated benefits from and barriers for implementing mental health specialist video consultations

Spontaneously reflecting on potential solutions for the aforementioned challenges in current care, some participants, namely three clinicians/representatives of their professional associations, one representative of a government agency, and one payer, anticipated especially integrated care models to address these structural problems regarding current treatment of patients with mental disorders.

*"However, the communication between mental health specialists and GPs is definitely very poor. (...) So that would be the main task: merely improving the communication." (clinician/representative of his/her professional association)*

In addition, some participants proposed optimisation of clinical workflows such as diagnostics, and access to psychotherapy (two clinicians/representatives of their professional associations, one payer, one health care policy maker), as well as online interventions (two clinicians/representatives of their professional associations, one representative of a government agency) as possible solutions. The introduction of consulting hours for patients with more acute distress or an increase of the number of available mental health specialists was rarely mentioned.

*"I think we always have to think of these services in terms of a stepped-care approach. Hence, we need different things, people are different; they have different needs. And that's why I also see a place for these online interventions in such a setting. They should be available when needed." (clinician/representative of his/her professional association)*

Furthermore, participants anticipated benefits and barriers mainly at the micro level. Specifically, participants mentioned benefits primarily for GPs and patients, and hardly any benefits for mental health specialists. The main benefit mentioned for GPs was relief by saving time resources and the possibility to refer patients to psychosocial treatment timely.

*“We are now using new technologies in patient care that were not available to us 20 years ago. Now they are here and now we must use them sensibly. And I believe that they are fully applicable and can also relieve [the GP of] some burden. Then, the GP will have time resources again or an appropriate work-life balance.” (*Health care policy maker*)*

Whereas the expertise of the GPs was generally not questioned, two payers cited that mental health specialists video consultations could lead to an improvement of GPs’ competencies in terms of diagnostic validation. For patients, all expert groups anticipated low-threshold access to specialist psychosocial treatment as main benefit. Moreover, some health policy experts (two clinicians/representatives of their professional associations, one payer, one health care policy maker) mentioned less stigma experience for patients and the possibility of rapidly clarifying the eligibility for care.

*“[...] many mentally ill people first see their GP. And often they actually stay there for a long time. In this respect, it is of course desirable that there is quicker access to other treatment options. It is also desirable to have the possibility to clarify the eligibility for care in the first place.” (Payer)*

Notably, participants rarely stated that the relationship of trust between the patients and GPs and the GP himself may function as a motivator for patients to engage with mental health specialists video consultations and thus provide a higher commitment (one clinician/representative of his/her professional association, one payer). In contrast, for mental health specialists, hardly any benefits were expected. However, as mentioned by some health policy experts (two clinicians/representatives of their professional associations, one payer, one representative of a government agency) potential benefits for mental health specialists could be workload relief by a more flexible arrangement of the place of work (e.g., providing consultations from home). In accordance with the few perceived benefits for the mental health specialists, they were anticipated to have a negative attitude towards mental health specialists video consultations by some health policy experts (three clinicians/representatives of their professional associations, one health care policy maker, one payer). Moreover, some participants expected a negative effect on the therapeutic alliance when using mental health specialists video consultations because they anticipated a lack of personal interactions and nonverbal cues compared to same-room treatment (five clinicians/representatives of their professional associations, one representative of a government agency). Additional workload for conducting mental health specialists video consultations and impact on clinical effectiveness were rarely discussed but still mentioned by one health care policy maker and one clinician/representative of his/her professional association. Notably, barriers regarding the patient-provider relationship were not mentioned for GPs. Perceived barriers for GPs included the requirement of a designated room and a stable internet connection and the organisation of a video consultation which might entail additional workload for the GPs and their medical assistants.

*“I just can't imagine it. I have a practice here […] and usually we run it with two assistants. Now someone comes and I initiate a video consultation. Where am I supposed to put him alone? They need a quiet atmosphere. Maybe he even needs some kind of supervision, an assistant, or the doctor to check if everything is all right. He must have the possibility to interrupt [the consultation] if necessary. So, from an organisational aspect it's ambitious, but maybe it can be tried” (clinician/representative of his/her professional association)*

GPs as well as patients were generally described as open-minded. Nevertheless, rejection by GPs was anticipated by one payer and almost all expert groups mentioned that the patient’s openness towards the model might dependent on his or her age or level of scepticism towards technology.

*"It varies. Well, we live here in a rural region. I could imagine that the patients are not quite as open-minded [towards mental health specialists video consultations] as in a university town. So of course, the patients will be rather young, yes? So, I don't think that a 70-year-old depressed patient is going to deal with this medium [video conferencing] now. But a 40-year-old burnout patient will.” (clinician/representative of his/her professional association)*

### Practical and regulative preconditions for the implementation of mental health specialists video consultations

In terms of regulative preconditions, all expert groups highlighted the importance to ensure information privacy (data protection) and data security. To ensure data protection and security, one clinician/representative of his/her professional association stated that GPs and their staff need to be trained to comply with it. Almost all expert groups expected no or only minor barriers in terms of legal regulations. While the representative of a government agency and two payers saw no need for legal changes, two clinicians/representatives of their professional associations and one health care policy maker were uncertain whether modifications regarding the delivery of mental health services were necessary. Furthermore, two health care policy makers stated that mental health specialists video consultations should not take place at home, but at the general practice or at other healthcare facilities, such as nursing home.

*"Of course, this [the consultations] also require data protection, data security and so on. So especially with such video-based services, this must of course have the highest priority." (Payer)*

Regarding payment regulations, health policy experts stated that contribution of GPs and mental health specialists must be reflected in adequate payment. All expert groups stated that GPs should receive a fixed payment for the provision of the necessary infrastructure (spatial and technical) and payment of mental health specialists should be equivalent to treatment as usual. Moreover, one clinician/representative of his/her professional association mentioned that an improved payment could lead as incentive to offer mental health specialists video consultations.

Practical preconditions for the implementation of mental health specialists video consultations refer to the development of networks, patient education, and open-mindedness. First, two clinicians/representatives of their professional associations and one payer emphasised the importance of establishing collaborations. All parties involved should get known to each other, roles should be clearly assigned, and procedures defined.

*"Well, I would even specifically incentivize the consultation between the GP and the mental health specialist such that in the run-up to the consultations the GP says, ‘This is Mr so and so, with him I have such and such problems’ but also that the mental health specialist provides a follow-up to the GP, at least, at the end of the process.” (clinician/representative of his/her professional association)*

Second, two payers and one patient representative considered detailed patient education to be essential, e.g., transparency regarding the procedure and data processing. One payer considered standardised assessments to be a helpful supplement and patient representatives an equivalent duration to guideline psychotherapy (50 min) as advisable. Finally, almost all groups of participants stated, that openness to new technologies such as mental health specialists video consultations is necessary for successful implementation. Success would essentially depend on the willingness of the providers to implement mental health specialists video consultations. Some clinicians/representatives of their professional associations assessed the openness towards online interventions very differently (e.g., ambulant mental health specialists would not be interested), openness in general would increase and providers more open to online interventions would already see advantages for themselves. Moreover, the innovation itself would have to be convincing and the benefits clearly recognisable.

## Discussion

### Principal results

The purpose of this pre-implementation study was to examine the perspective of health policy experts on (1) current challenges for continuity of care, (2) anticipated benefits and barriers of mental health specialists video consultations along with (3) practical and regulative preconditions for their sustained implementation into primary care.

First, regarding current challenges for continuity of care all participants mentioned similar problems, namely waiting times, number of available mental health specialists, and insufficient collaboration between inpatient and outpatient treatment as well as between ambulant GPs and mental health specialists.

Second, after describing the PROVIDE intervention, all participants mentioned similar benefits and barriers for the involved parties (GPs and their staff, mental health specialists, and patients) from mental health specialists video consultations. Overall, all participants mentioned benefits mainly for GPs (relief through saving time resources and the possibility to refer patients to psychosocial treatment) and patients (low threshold access). In terms of barriers, almost all expert groups expected negative effects on the therapeutic relationship when conducting mental health specialists video consultations. Lack of a designated room and a stable internet connection was mentioned as main barriers for GPs.

Finally, the provision of increased collaborations and data protection were considered as preconditions for sustained implementation of mental health specialists video consultations.

### COVID-19 as a facilitator for eHealth implementation

We collected data before the COVID-19 pandemic in Europe. As response to nationwide lockdowns and curfews and to minimise the risk of infection in face-to-face settings, health care systems tried to treat patients remotely via eHealth and especially video-based treatment options wherever possible. Consequently, eHealth treatments in general and mental health specialists video consultations in particular have been implemented rapidly to some degree already which often has been the only possible way to provide adequate continuous treatment and has been facilitated by the providers’ motivation to rapidly adapt to patients’ limited access to mental health care [52–55].

Providers as well as patients accepted the new modes of delivery and tried to continue their treatments as best as they could. Depending on the pre-COVID conditions that prevailed within each respective health care environment, success of the transition to remotely delivered care varied. For example, the Veteran Health Administration in the USA have had a focus on mental health specialists video consultations long before the COVID-19 pandemic. Consequently, the expansion to more video supported treatment was relatively seamless [56, 57]. In Australia, by quickly adapting reimbursements schemes, providers and patients rapidly adopted mental health specialists video consultations and the number of video supported psychological or psychiatric sessions increased sharply [58, 59]. However, since most health care systems were not prepared for this emergency, legal and regulatory frameworks and reimbursements schemes have often been changed without the usual path of consensus building within regulatory and legislative authorities [60–62].

This leads to open questions regarding the comprehensive implementation of eHealth interventions and mental health specialists video consultations after the COVID-19 pandemic, when it will be possible to approach physicians in person again. Therefore, our findings will inform the process of future implementation of mental health specialists video consultations from the perspective of health policy experts.

### Comparison with prior work

In general, participants expected mental health specialists video consultations to be able to improve the care of primary care patients with mental health problems. Attributing this to a more timely access to psychosocial treatment is congruent with the findings of a large survey study investigating health policy experts’ expectations of digital treatment for depression from eight European countries [39]. While in the survey this potential benefit was only the second most important, the most important benefit of the implementation of digital treatment for depression was the reduction of treatment costs. In our study, this aspect was not discussed at all. In general, in our study benefits were mainly seen on the micro level instead of the macro level which would have included economic effects of the implementation of mental health specialists video consultations which is an interesting observation given the participant group.

Regarding potential barriers, survey participants assessed their respective health system not ready for the implementation of digital treatment [39]. Again, in our study potential barriers were seen on the micro level such as negative effects on the patient-provider relationship or reservations towards mental health specialists video consultations on the side of mental health specialists. These was also mentioned in the survey but considered not as important as in our study.

Generally, our small sample does not allow for any inferences concerning the pattern of health policy experts to locate benefits on the micro- rather than the macro-level. However, it seems somewhat plausible that health policy experts did not consider cost advantages because some of them were unsure about current reimbursement schemes and legal frameworks pertaining to video consultations.

Our findings on barriers and benefits are also in accordance with those from a systematic review which included expert discussions from two international informatics conferences and a systematic literature review [63]. In this review, authors present health policy experts’ perspectives on implementation of eHealth services in general but not video consultations for mental health care specifically. However, the barriers and benefits which were identified in our interviews match with the findings from the review. Furthermore, several benefits and barriers were attributed similar importance to, such as increasing collaboration or lack of eHealth knowledge which requires comprehensive training for users. To account for the need for stronger collaboration between GPs and mental health specialists, several core components featured in two clinical trials we have recently embarked on focus on remote collaboration: structured electronic feedback concerning the diagnostic assessment of the mental health specialist, real-time outcome monitoring of the mental health specialists video consultations the results of which are forwarded to the GP, and a shared written treatment plan negotiated between the patient, the specialist, and the GP [64, 65].

Almost all participants in our study considered that mental health specialists video consultations would have a negative effect on the patient-provider relationship attributing this to a lack of nonverbal cues and interactions. Although this might be true for the clinician’s perspective, for patients the online setting might be empowering since power differentials may be overcome [65]. In general, eHealth interventions can engage and empower patients to actively participate in the treatment process by providing more information about their treatment and consequently feel more confident when it comes to conversations with the provider [66]. In a pilot study on a telemedicine medication counselling intervention for young HIV-positive African Americans, patients viewed the intervention as convenient and efficient and beneficial for their knowledge and the relationship with their provider because of the less intimidating nature of the dialogue [67].

Furthermore, in an implementation study both patients with depression and mental health clinicians reported that their relationship improved by using an accompanying comprehensive online tool including medication and appointment reminders, as well as psychometric evaluations on a regular basis, as an adjunct to their usual psychotherapy sessions [68]. However, effects of mental health specialists video consultations on the relationship between the patient and the mental health specialist in particular have hardly been investigated and require further research, from which guidelines could subsequently be derived [69]. In video based integrated care models, patients are referred directly by their GPs and their often more intimate relationship may function as a facilitator to improve the relationship between the patient and the mental health specialist during the mental health specialists video consultations although in our study this aspect was hardly ever discussed.

Health policy experts highlighted data protection and data security as main requirement, users hardly ever discuss compliance with these. This is not surprising, as health policy experts must ensure and monitor compliance with data protection and security at regulatory level. In contrast, users (GPs, mental health specialists and patients) tend to have less concerns about data protection and security [70]. However, data privacy protection will always be an essential prerequisite for the implementation of video consultations and although quiet and confidential places for video consultations within primary care practices may often be available [44] and German government regulations require adherence to the EU General Data Protection Regulation from certified operators (e.g., video and audio communication are not recorded or stored on any server), data breaches can never fully be ruled out for video consultations.

Openness to eHealth interventions is essential for successful implementation. Several studies showed that GPs and mental health specialists are generally open towards video-based treatment models, although concerns regarding the therapeutic alliance between the patient and the mental health specialist and an increased workload for the GP are entertained [39, 42, 71–73]. Our study adds, that to foster the openness there should be an appropriate payment for both the GP for provision of infrastructure and the mental health specialist for conduction of the actual video consultation. During the COVID-19 pandemic, reimbursement schemes have been modified and should be permanently extended to ensure a consistent openness on the providers’ end.

### Future work involving health policy experts

The digitalisation of treatment provision during the COVID-19 pandemic leads to open questions for implementing mental health specialists video consultations in the future and in a post COVID-19 time [74]. Will mental health specialists video consultations be applied more broadly and more often? Will the current changes in legal frameworks and reimbursement schemes stay in place?

In answering these questions and providing solutions health policy experts will play a crucial role. They will have to analyse the lessons learned from the pandemic and transpose them into recommendations for implementation and application [75–77]. Recent reports suggest that eHealth interventions may be useful for specific situations and demands, such as the provision of a timely initial appointment. For other aspects, they may be not appropriate or even unfavourable (e.g., negative effect of patient-physician relationship) [78, 79].

It will be up to health policy experts to determine, in which situations eHealth interventions in general and mental health specialists video consultations in particular can be of value for (mental) health care. In doing so, an overarching recommendation and proposed principle is the inclusion and involvement of all significant stakeholders [80–83]. This will ensure a sustained implementation, even beyond the pandemic.

### Limitations

First, focus groups might have been more appropriate to discuss different problems and potential solutions from various perspectives of health care. However, for this kind of participants we anticipated that it would be nearly impossible to find a mutual date to conduct focus groups. Consequently, we chose individual telephone interviews and gained data from 15 different health care experts. This gave us the opportunity to get the individual perspective without being compromised by social desirability often occurring in focus groups.

Second, we did not provide any socio demographic information on the participants. Although this might have made our findings more meaningful, we decided only to present institutional information on the participants. By adding demographic data, we could not have rule out, that these health policy experts could be identified, since some of them were high-level representatives for the respective expert group (e.g., chairwomen and chairmen of large insurance companies, government employees).

Finally, our sample consisted of experts from different health policy fields. Therefore, it was relatively heterogeneous. This may limit the generalisability of the results as some of them represented opposing stakeholder groups (e.g., clinicians and payers). However, sampling such different groups of stakeholders gave us the opportunity to cover multiple perspectives which supports the generalisability for the group of health policy experts in general. This would have been much more difficult if we had focused on only one group. Still, within the expert groups the number of participants was relatively low.

## Conclusion

From the health policy experts’ perspective, mental health specialists video consultations may potentially contribute to overcoming current problems in the care for mental health patients. By promoting integrated care models, intersectoral communication and collaboration can be improved which may lead to more accessible mental health care.

### Implications

The COVID-19 pandemic revealed that many health care systems were not very well prepared to provide remotely delivered treatment. To address these challenges in the future and implement mental health specialists video consultations sustainably, it will be crucial for health policy experts to involve all relevant stakeholders in the development and monitor the implementation process to be able to address raising barriers. This will contribute to decisions on the application of eHealth interventions and mental health specialists video consultations in the future. One main barrier are still technical conditions. Prior to implementation, health policy experts should make sure that the technical environment is sufficient for the conduct of mental health specialists video consultations. Besides technical conditions (e.g., internet access, available hardware), focus should be on concerns regarding data security and reimbursement schemes. A secure data connection between the users is fundamental and improves providers’ trust, openness and consequently readiness to implement. When these aspects are considered, mental health specialists video consultations can be a valuable addition and an easier access to mental health care, especially for patients who are usually hard to reach. Furthermore, GPs’ and mental health specialists’ awareness and knowledge about mental health specialists video consultations as a viable alternative to same-room treatment should be improved by implementing comprehensive trainings and information strategies on the appropriate use of mental health specialists video consultations. This would encourage GPs and mental health specialists to further engage in video consultations in routine care.

## Supplementary Information

**Additional file 1:.** Search Strings for the Systematic Review.

**Additional file 2:.** Interview Guide for Health Policy Experts.

**Additional file 3:.** Overview of the key themes, including definitions and supporting quotes.

## Data Availability

All data generated or analysed during this study are included in this published article and its supplementary information files.

## Abbreviations

GP: General practitioner
GPs: General practitioners
