# Supplementary material for: Health policy experts’ perspectives on implementing mental health specialist video consultations in routine primary care – a qualitative interview study
Source: BMC Health Serv Res. 2021 Jul 20;21:713. doi: 10.1186/s12913-021-06676-x (PMC8293503; doi:10.1186/s12913-021-06676-x)
Supplement: Supplementary file 1 — Additional file 1:. Search Strings for the Systematic Review. [file 12913_2021_6676_MOESM1_ESM.docx]

**Additional File 1. Search Strings for the Systematic Review**

**MEDLINE:**

**Filters:** Fields: Title/Abstract; Species: Humans; Languages: English, German

**Search string (including filters):**

("policy makers"[Title/Abstract] OR "policy-maker"[Title/Abstract] OR "policy making"[Title/Abstract] OR "health policy experts"[Title/Abstract] OR stakeholders[Title/Abstract] OR "health policy"[Title/Abstract] OR expert*[Title/Abstract]) AND ("mental disorders"[MeSH Major Topic] OR "psychiatry and psychology category"[MeSH Major Topic] OR "psychotherapy"[MeSH Major Topic]) AND ("telemedicine"[MeSH Major Topic] OR "virtual care"[Title/Abstract] OR "video consultation"[Title/Abstract] OR "video consultations"[Title/Abstract] OR "video consult*"[Title/Abstract] OR "videoc*"[Title/Abstract] OR "video c*"[Title/Abstract] OR "telepsy*"[Title/Abstract] OR "telemental"[Title/Abstract] OR "E-mental"[Title/Abstract]) AND "humans"[MeSH Terms] AND ("German"[Language] OR "English"[Language])

**Link:** https://pubmed.ncbi.nlm.nih.gov/?term=%28%22policy+makers%22%5BTitle%2FAbstract%5D+OR+%22policy-maker%22%5BTitle%2FAbstract%5D+OR+%22policy+making%22%5BTitle%2FAbstract%5D+OR+%22health+policy+experts%22%5BTitle%2FAbstract%5D+OR+stakeholders%5BTitle%2FAbstract%5D+OR+%22health+policy%22%5BTitle%2FAbstract%5D+OR+expert*%5BTitle%2FAbstract%5D%29+AND+%28%22mental+disorders%22%5BMeSH+Major+Topic%5D+OR+%22psychiatry+and+psychology+category%22%5BMeSH+Major+Topic%5D+OR+%22psychotherapy%22%5BMeSH+Major+Topic%5D%29+AND+%28%22telemedicine%22%5BMeSH+Major+Topic%5D+OR+%22virtual+care%22%5BTitle%2FAbstract%5D+OR+%22video+consultation%22%5BTitle%2FAbstract%5D+OR+%22video+consultations%22%5BTitle%2FAbstract%5D+OR+%22video+consult*%22%5BTitle%2FAbstract%5D+OR+%22videoc*%22%5BTitle%2FAbstract%5D+OR+%22video+c*%22%5BTitle%2FAbstract%5D+OR+%22telepsy*%22%5BTitle%2FAbstract%5D+OR+%22telemental%22%5BTitle%2FAbstract%5D+OR+%22E-mental%22%5BTitle%2FAbstract%5D%29+AND+%22humans%22%5BMeSH+Terms%5D+AND+%28%22German%22%5BLanguage%5D+OR+%22English%22%5BLanguage%5D%29&sort=

**No. of records:** 597 (as of April 28^th^, 2021)
