# Supplementary material for: Health policy experts’ perspectives on implementing mental health specialist video consultations in routine primary care – a qualitative interview study
Source: BMC Health Serv Res. 2021 Jul 20;21:713. doi: 10.1186/s12913-021-06676-x (PMC8293503; doi:10.1186/s12913-021-06676-x)
Supplement: Supplementary file 2 — Additional file 2:. Interview Guide for Health Policy Experts. [file 12913_2021_6676_MOESM2_ESM.doc]

**Additional File 2. Interview Guide for Health Policy Experts**

*(Finalized version as of December 2017)*

**A. Current health care of patients with mental health conditions in primary care**

- Considering outpatient services, what do you think of the current health care of patients with mental health conditions?
  - What would you describe as **distinctive features** of health care delivery for patients with mental disorders?
  - To what extent do you perceive gaps in the current health care system?
- Which **measures** aiming at improving the quality of mental health care do you conceive as reasonable?

**B. Introduction of the PROVIDE intervention model, implementation potential und compatibility (e.g. readiness for change)**

- Do you have any further questions concerning the intervention model?
- Would you be willing to vote in favor for **and support the treatment** model outlined above?
- What **legal and social regulations** do videoconsultations as envisaged in the PROVIDE project have to meet?
- What **problems/risks/challenges** come to your mind in regard to feasability, practicability and sucess of treatment
  - for patients
  - for medical assistants and health care personnel (e.g., administrative burden)
  - from the perspective of health politics (e.g., administrative burden)
- What do you consider as potential **advantages**?
  - for patients
  - for medical assistants and health care personnel
  - from the perspective of health politics
- Which models of financial remuneration do you consider appropriate?
- What are **preconditions for a nationwide implementation** of the treatment model?
  - Do you think a nationwide implementation is **realistic**?

**C. Interview termination**

- Are there any remaining aspects important to you that we have not addressed at this point?
- Do you have any further questions?
- May we contact you again when conducting any surveys and/or studies in future?
- Can you think of any other health policymakers who might be interested in the topic?
