# Supplementary material for: Health policy experts’ perspectives on implementing mental health specialist video consultations in routine primary care – a qualitative interview study
Source: BMC Health Serv Res. 2021 Jul 20;21:713. doi: 10.1186/s12913-021-06676-x (PMC8293503; doi:10.1186/s12913-021-06676-x)
Supplement: Supplementary file 3 — Additional file 3:. Overview of the key themes, including definitions and supporting quotes. [file 12913_2021_6676_MOESM3_ESM.docx]

**Additional File 3. Overview of the key themes, including definitions and supporting quotes**

| ***Key theme*** | ***Definition*** | ***Subthemes*** | ***Supporting quotes*** |
| --- | --- | --- | --- |
| Current Practice - macro level perspective | | | |
| Current Challenges for Continuity of Care | Participants perception of the current care of patients with mental disorders in terms of distinctive features and gaps. | Regulations | 1. Waiting times:   "[...] And of course the first reason is waiting times. Basically, [...] we have the situation that, in principle, psychotherapy is accessible to almost all citizens due to the financing by the health insurance funds. But the waiting times are partly enormous, they are very different, urban, rural areas and so on. So that is actually where the shoe pinches the most from the patient's point of view." (PA, Participant 01)   1. Insufficient number of available mental health specialists:   “However, we still see a shortage, (...) especially when it comes to guideline treatment, but we assume that there may also be shortages for acute treatment, or will be, because we simply know that it varies greatly from region to region, that the need for care is higher than the current supply of psychotherapy.” (CPA, Participant 03) |
|  |  | Fragmentation of sectors/Insufficient collaboration | “[...] what from our experience of the last few years still represents a substantial problem in the care of mentally ill people is the still existing strong fragmentation. That means that we have a very closed inpatient area and the outpatient area. [...] So that, it is still often difficult to actually bring together the different participants in the treatment process.” (P, Participant 15) |
|  |  | Limited equity of care | “[...] A lot of resources, time and manpower are invested in the psychotherapy of rather mild disorders. And the more seriously disturbed, especially psychiatric (...) patients have great problems getting appropriate care. [...] Now you have to differentiate again for certain illnesses or disorders. But especially members of lower social classes people with language problems and migration background, also older patients, tend to be cared for worse, inadequately. While patients from the academic milieu, from the upper 20 per cent of our society, actually receive good care in general.” (CPA, Participant 06) |
| Suggested solutions | Health policy experts’ anticipated solutions to overcome limiting aspects of care of patients with mental disorders | Cross-sectoral and cross-professional collaboration/Integrated care models | "But I would also define [intersectoral cooperation] as something a bit different. Not just referral, but something that goes beyond that. So actually, a stronger networking of the disciplines. [...] Because, at least in my perception, it is often difficult, because sometimes one practitioner doesn't know anything about the other. And because of that, this process of treatment is sometimes very fragmented, especially because with mental illnesses the whole process often goes on over a long period of time. Often over years. And to that extent it is also very much about establishing structures, of course. That enables stronger networking between those involved in treatment.” (P, Participant 15) |
|  |  | Diagnostics, classification, access to psychotherapy | “One topic that we are now also discussing more intensively with the GPs is how they can look at their patients for mental illness issues in their practices in a more structured way.” (P, Participant 15) |
|  |  | Online interventions | "And there [the problem of waiting times; equity of care] I see a need for improvement so that everyone can reach psychotherapy equally, so to speak, and telemedicine services could certainly contribute to breaking down these barriers.” (GA, Participant 08) |
|  |  | Acute consulting hour | “I believe that [improving the quality of care] cannot be regulated with interventions. In the meantime, psychotherapists are obliged to provide acute consultations. (...) We'll see if that works in the long term. (...) But it would certainly make sense if everyone who treats mental illnesses had appointments reserved, let's say for one hour a day, where an acute patient can also drop in.” (CPA, Participant 14) |
|  |  | Number of available mental health specialists | [...] Within the system [of mental health care] (...) the major problem is actually that there is far too little manpower and financial power to really get close to the huge issue of mental illness. Yes. That's why we need more [psychotherapy] places. [...].” [CPA, Participant 09] |

| ***Key theme*** | ***Definition*** | ***Subthemes*** | ***Supporting quotes*** |
| --- | --- | --- | --- |
| Video consultations in the GPs practice - Expectations regarding MHSVC | | | |
| Benefits of MHSVC for GP | Anticipated benefits regarding MHSVC for the GP and the medical assistants | Relief | “All right, but then there is certainly a part of patients who go to the GP and the GP is of the opinion that these should now - because it is now the third depressive episode within a year - look deeper and the GP does not have enough time for these patients to make an offer to talk and perhaps he does not have the tools for this. Then I can very well imagine a telemedical consultation.” (HPM, Participant 07) |
|  |  | Skills development | “I simply assume that the competence of the GP increases at this point and the accuracy of the assessment also increases. Is this a patient who is suitable for it [video consultation]? Is there a need for such a video consultation? Or is it perhaps a patient that I have to refer directly to a specialist practice, where a different level of treatment has to be provided? [P, Participant 10] |
| Barriers of MHSVC for GP | Anticipated barriers regarding MHSVC for the GP and the medical assistants | Resources | “[...] I could also imagine that this question of spaces, of infrastructure, is indeed a problem.” (P, Participant 05) |
| Benefits of MHSVC for Patients | Anticipated benefits regarding MHSVC for patients | Low-threshold access | "[...] This could actually be a chance to get more people with mental disorders at an early stage before we see so many chronic conditions. Because it is obvious that the more chronic it becomes, the more severe it becomes and the longer it will take to treat. So that's where I see an opportunity to simplify access in that direction." [CPA, Participant 02] |
|  |  | Lowering of the inhibition threshold/decreased stigma experiences | “[...] the hurdles to seek psychotherapeutic advice are, I think, even greater among the rural population than among the urban population. (...) So in the rural areas, when a neighbour finds out that someone else has been to a psychotherapist, it's different from when you go anonymously in the city. So, I think that in the rural areas there could also be an additional advantage, that the social barrier is gone.” [CPA, Participant 04] |
|  |  | Clarification of needs | "Whenever it is a matter of diagnosing mental illnesses faster and more precisely from the primary care sector and identifying the need for treatment, this actually only brings advantages for all those involved.” [P, Participant 05] |
|  |  | Relationship of trust between GP and patent | “We have a treatment in a familiar environment. That is certainly something that is important for the patient, that he also has the feeling that his general practitioner is also involved to some extent in the treatment and also plays a role there.” (P, Participant 10) |
| Barriers of MHSVC for patients | Anticipated barriers regarding MHSVC for patients | Scepticism/rejection | "There will be patients who don't want to [...] who simply will never trust technology. Especially older patients. They won't be reached at all, but in this respect (...) I would not only rely on the current system, but I would say that we should try something like this (...)" [GA, Participant 08]. |
| Benefits of MHSVC for mental health specialists | Anticipated benefits regarding MHSVC for mental health specialists | Relief | “For the psychotherapist, it is perhaps really a possibility to use one's own resources in a different way and perhaps to reach more patients if I take this low-threshold treatment path at that point. Therefore, I could imagine that psychotherapists would be interested in doing this because, as I said, I can treat patients in a lower-threshold way. Maybe I can reach them earlier. I can reach more patients.” [P, Participant 10] |
|  |  | Flexibility | “[...] Perhaps this is also an interesting option for psychotherapists who want to work from home and do not want to or cannot set up a practice.” [CPA, Participant 06] |
| Barriers of MHSVC for mental health specialists | Anticipated barriers regarding MHSVC for mental health specialists | Scepticism/rejection | “And this is what I am generally trying to convey to my colleagues at the moment, that the use of the Internet in treatments should not abolish the practice in the long term, but rather that complementary possibilities should be tested and investigated. [...] If you work as a psychotherapist in a practice and know that the whole is paid by the health insurance company, then I can understand the fear [...] that the insurance companies are promoting this in order to replace psychotherapists in the long term.” [CPA, Participant 03] |
|  |  | Additional effort | “The benefit does not belong to the psychotherapist, on the contrary. The psychotherapist has more effort. He has to afford the technical effort. Usually, he also has to deal with other technology and other processes.” (HPM, Participant 13) |
| Mode of delivery | Differences between face-to-face contact and video consultation | Therapeutic alliance | “I think that a general concern is that I do not have direct, immediate contact with the patient. And you have to take that seriously. So certain things of the patient are no longer as good, or perhaps not even perceptible.” [CPA, Participant 03] |
|  |  | Effectiveness | "(...) I personally prefer a direct contact, of course, and if you now say that you do it in [city], then I find the video consultation doubtful. I might find that quite good as a gateway, but if the [psychotherapist] is sitting three streets away, it is of course not comparable in terms of effectiveness with a so-called face-to-face contact." [CPA, Participant 12] |

| ***Key theme*** | ***Definition*** | ***Subthemes*** | ***Supporting quotes*** |
| --- | --- | --- | --- |
| Practical and regulative preconditions | | | |
| Requirements regarding the implementation of MHSVC | Requirements that must be met for MHSVC | Data protection and data security | “(...) all the formal conditions, data protection etc., that's obvious anyway, that has to be guaranteed.” (CPA, Participant 03) |
|  |  | Legal regulations | “(...) with the e-health act there is already such a regulation, where exactly such things are explicitly desired and should be promoted. And in terms of social law, it is finally all part of the normal treatment, through remuneration, EBM numbers and so on. That, of course, is probably debatable.” (P, Participant 05 |
|  |  | Setting | “But I would not consider psychotherapeutic or psychiatric treatment adequate in the home environment (...). As I said, data protection and setting alone are not suitable for it. Instead, it has to be a professional environment, but as I said, that would definitely be the case in a retirement and nursing home, for example.” (HPM, Participant 13) |
| preconditions for the implantation of MHSVC | Recommendations for action and suggestions for improvement for (further) development of the MHSVC approach | Payment | “The psychotherapist takes 45 minutes of his time. For him, it is actually no difference whether the patient sits in front of him or whether he looks at him via video chat. The therapist has to be paid for the hour of therapy. [...] In addition, he must receive a fixed payment for the secure technology, for the connection.” (P, Participant 07) |
|  |  | Collaboration | “I would also specifically encourage the, let's say, consultative connection between the GP and the psychotherapist. Both at the initiation, that the GP says, with this patient I have these problems. Medical history, medication, diagnoses, my suspicions. And also, that the psychotherapist gives feedback to the GP. I mean, at least at the end of this process.” (CPA. Participant 06) |
|  |  | Distribution of roles | “It is different in psychotherapy, where the focus is on the patient and the GP does not have to be present. He can't contribute anything either. Maybe during the anamnesis, during the first consultation. But otherwise he must not disturb the relationship between therapist and patient.” (HPM, Participant 07) |
|  |  | Open-mindedness | "I guess whoever's doing this is open-minded. You'll never get every psychotherapist and GP to open up to a procedure like this. And those who do, I assume they see more opportunities than risks." [CPA, Participant 04] |
|  |  | Patient education | “(...) to explain the patient what they are doing and how data is being processed. In any case, that is always a very important quality feature, that the user can consciously decide - yes, I want to do this. And it is also clear to me [as a patient] what happens with my data. Or, if necessary, they can also consciously decide against it.” (P, Participant 05) |
